# Intradialytic hypotension and cardiac remodeling: should we consider the renin-angiotensin-aldosterone system?

**DOI:** 10.1080/0886022X.2021.1901741

**Published:** 2021-03-29

**Authors:** Juanli Wang, Wenjun Zhang, Yuanyuan Qi

**Affiliations:** Department of Nephrology, Second Hospital of Lanzhou University, Lanzhou, PR China

Dear Sir,

We have read with great interest the paper entitled ‘High ultrafiltration rate induced intradialytic hypotension is a predictor for cardiac remodeling: a 5-year cohort study’ by Yu et al. [1]. It is a retrospective cohort study that was firstly testified the contribution of intradialytic hypotension (IDH) to cardiac remodeling in the clinical base. A total of 209 hemodialysis (HD) patients (118males and 91 females) with an average age of 52.92 ± 18.68 years were collected. 96 cases of IDH (≥4 hypotension events/3 months) and 113 cases without IDH (<4 hypotension events/3 months). Compared with those with no-IDH, IDH-prone patients were older, had higher BMI, interdialytic weight gain and ultrafiltration rate, lower predialysis and postdialysis BP (*p* < .05). In the IDH group, decreased ejection fraction, larger left atrium diameter index as well as larger left ventricular mass index (LVMI) (*p* < .05, *p* < .01) were observed at the end of the follow-up than at the recruitment time. In multivariate logistic model, the interaction between ultrafiltration rate (UFR) and IDH was notably associated with LVMI variation (*OR* = 1.37). High UFR (>10 mL/h/kg) would cause cardiac remodeling only in IDH-prone patients. High UFR induced IDH is a determinant of cardiac remodeling. I pay special attention to the results of this research because it caused me some confusion.

**Figure 1.**
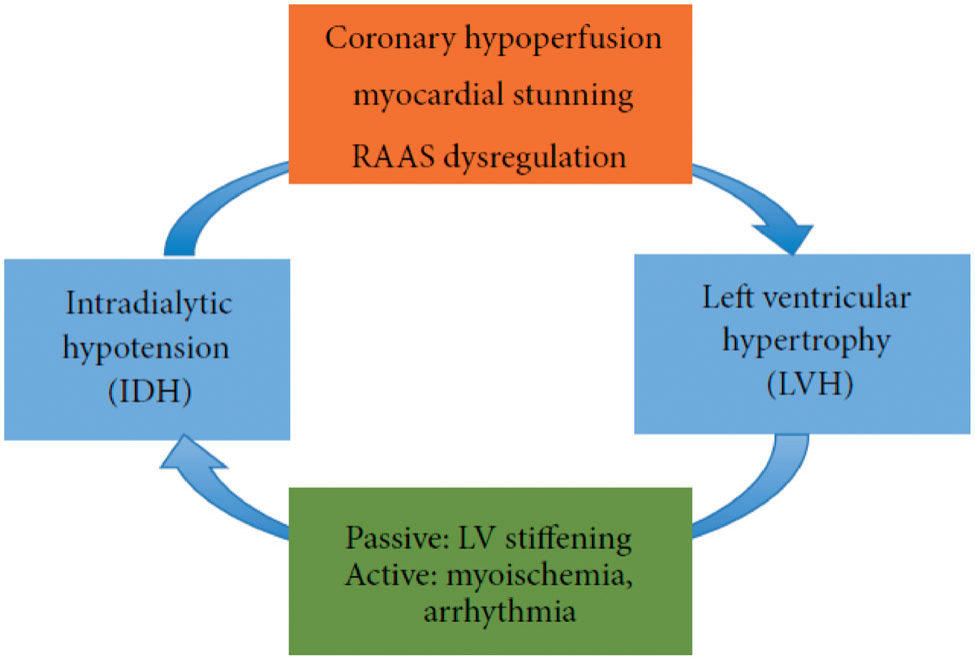
Diagram illustrating the interaction between IDH and LVH. (From Biomed Res Int. 2015; 2015:724147.).

As shown in Figure 1, the occult effect of IDH in the development of left ventricular hypertrophy (LVH) encompasses three aspects. Coronary hypoperfusion and myocardial stunning as important contributors of cardiac remodeling in HD patients have been discussed, but the renin-angiotensin-aldosterone system (RAAS) dysregulation have not been involved in the original article by Yu et al. The RAAS is over-activated in chronic kidney disease with patients having inappropriately elevated aldosterone production relative to their fluid status [2]. Several studies have found that IDH-prone patients have higher baseline plasma angiotensin II levels than those who are IDH-resistant, and activation of the RAAS is known to be involved in LVH development, independent of afterload [3,4]. Spironolactone is as a represent therapeutic strategy for aldosterone antagonism. In an open label trial of spironolactone in Japanese patients treated with peritoneal dialysis, spironolactone prevented LVH and preserved left ventricular ejection fraction [5].

Nesarhosseini et al. [6]. conducted a case-control study involving 69 ESRD patients on dialysis with an average aldosterone level of 152.95 ± 81.25, divided into the case group (*n* = 52, exhibiting LVH) and the control group (*n* = 17, no ventricular hypertrophy observed in the echocardiography). Angiotensin-converting enzyme inhibitors (ACEI) and angiotensin-receptor blocker (ARB) drugs were substituted with other medications for 2 weeks in hypertensive patients. It definitely shows that serum aldosterone level is significantly associated with LVH in ESRD patients and is a predictor of LVH. A prospective observational trial of 27 HD patients showed that LVH was associated with aldosterone levels independently of confounding factors [7].

Although the conclusion of the original article is consistent with some previous studies, it ignores the detection and analysis of important indicators in RAAS, and there is no information about specific interventions and adjustment of antihypertensive medication. In conclusion, IDH and cardiac remodeling, especially LVH, are closely related to each other. A clear understanding of the complex interactions between IDH and LVH might assist in devising useful strategies. The inclusion of RAAS will further enhance the importance of this study.
